# A qualitative exploration into the presence of TB stigmatization across three districts in South Africa

**DOI:** 10.1186/s12889-023-15407-2

**Published:** 2023-03-15

**Authors:** Daniel DeSanto, Kavindhran Velen, Richard Lessells, Sewele Makgopa, Dumile Gumede, Katherine Fielding, Alison D Grant, Salome Charalambous, Candice M Chetty-Makkan

**Affiliations:** 1 Africa Health Research Institute, KwaZulu-Natal, South Africa; 2 The Aurum Institute, Johannesburg, South Africa; 3 School of Public Health, Faculty of Health Sciences, University of the Witwatersrand, Johannesburg, South Africa; 4 London School of Hygiene & Tropical Medicine, TB Centre, London, UK; 5 School of Laboratory Medicine and Medical Sciences, College of Health Sciences, University of KwaZulu-Natal, Durban, South Africa; 6 KwaZulu-Natal Research Innovation & Sequencing Platform, School of Laboratory Medicine and Medical Sciences, University of KwaZulu-Natal, Durban, South Africa; 7 Centre for General Education, Durban University of Technology, Durban, South Africa; 8 Health Economics and Epidemiology Research Office (HE2RO), Wits Health Consortium, Faculty of Health Sciences, University of the Witwatersrand, Johannesburg, South Africa

**Keywords:** Stigma, Tuberculosis stigma, HIV Stigma, Stigma interventions, Active case finding, Health seeking Behaviour, Health System strengthening

## Abstract

**Background:**

Tuberculosis (TB) stigma is a barrier to active case finding and delivery of care in fighting the TB epidemic. As part of a project exploring different models for delivery of TB contact tracing, we conducted a qualitative analysis to explore the presence of TB stigma within communities across South Africa.

**Methods:**

We conducted 43 in-depth interviews with 31 people with TB and 12 household contacts as well as five focus group discussions with 40 ward-based team members and 11 community stakeholders across three South African districts.

**Results:**

TB stigma is driven and facilitated by fear of disease coupled with an understanding of TB/HIV duality and manifests as anticipated and internalized stigma. Individuals are marked with TB stigma verbally through gossip and visually through symptomatic identification or when accessing care in either TB-specific areas in health clinics or though ward-based outreach teams. Individuals’ unique understanding of stigma influences how they seek care.

**Conclusion:**

TB stigma contributes to suboptimal case finding and care at the community level in South Africa. Interventions to combat stigma, such as community and individual education campaigns on TB treatment and transmission as well as the training of health care workers on stigma and stigmatization are needed to prevent discrimination and protect patient confidentiality.

**Supplementary Information:**

The online version contains supplementary material available at 10.1186/s12889-023-15407-2.

## Background

Tuberculosis (TB) remains a global public health crisis despite concerted control and eradication efforts. In 2020, the World Health Organization estimated 9.9 million incident cases of TB worldwide resulting in roughly 1.2 million deaths [1]. Diagnosis of people with TB and service provision have been disrupted since the start of the COVID-19 pandemic [2, 3]. South Africa, a country that accounts for 3.3% of annual incident cases globally, has had setbacks in TB case detection and the cascade of care [1].

TB stigma identifies individuals with TB as different. People with TB are assumed to possess negative character traits and are seen as responsible for their illness [4]. Stigmatization happens through the process of applying beliefs, attitudes, and behaviours to a member/potential member of a stigmatized group [4–6]. This legitimizes the need for others to identify, exclude, and protect themselves from people with TB [5, 7]. The manifestation and interpretation of TB stigma is contextually dependent, set against the backdrop of one’s community, but its presence is widely acknowledged and increasingly documented within public health research [8]. Further, TB public health messaging has been inconsistent at best, with the potential to indirectly contribute to stigmatization [5, 9]. The utilization of a conceptual model on stigma in health can align terminology and provide nuanced cross cutting perspectives into the processes of stigmatization [6].

Systematic reviews on TB stigma highlight positive associations between increased stigma and HIV, fear of being infected/infecting others, increase in TB diagnostic delay, and treatment noncompliance [10–13]. South African studies found that perceptions of stigma impacted how people with TB navigate clinical services [14, 15]. In communities across South Africa, TB stigma has been investigated alongside HIV stigma due to the contextual double burden of disease [16, 17]. Further studies found low self-worth amongst individuals with internalized stigma and that anticipated community-stigma impacts disclosure of one’s status due to the fear of negative reactions [18]. Community members identified coughing, weight loss, sweating, and diminished appetite as symptoms of TB, and/or HIV, contributing to stigmatization of symptomatic individuals [18]. Prior investigations into TB stigma in South Africa have predominantly explored TB stigma in the contexts of treatment, health service delivery, patient experience, stigma measurement, and evaluations of stigma reducing interventions. Understanding the drivers of stigma from the perspectives of community members can inform approaches to strengthen active case finding, treatment, and retention.

This qualitative investigation explores the presence of stigma from a community-level perspective, unearthing factors driving TB stigma and its impact on health seeking behaviour across three South African districts.

## Methods

This exploratory analysis was part of a larger qualitative study which examined the role of ward-based outreach teams in the implementation of TB household contract tracing [19]. All methods were carried out in accordance with relevant guidelines and regulations. While we did not explicitly set out to investigate stigma, TB stigma emerged as a dominant theme during the analysis. Transcripts from the larger qualitative study were utilized for this analysis therefore data collection methods are congruent across investigations. All transcripts were included except for semi-structured in-depth interviews with TB program stakeholders as they did not represent the perspectives of individuals living within the community. This analysis is situated within a social constructivist paradigm to explore the presence of TB stigmatization within the lives of study participants. All researchers from the larger qualitative study were involved in this analysis. Interviews and focus groups were conducted by co-authors, SM (Bachelor’s degree), CMC (PhD), KV (PhD), DG (PhD) and RL (MD), a mixture of male and female public health researchers with prior experience in qualitative research. None of the researchers were from the communities included in the study. Prior to the study there was no relationship between the research team and any study participants, except with community stakeholders who frequently engage with the health system and therefore had previous contact with the interviewer and note taker. No researcher characteristics surfaced that might have influenced research questions, methods, results and/or the transferability of this analysis.

### Sampling strategy & data collection

Study participants were sampled across three South African districts. All districts had a high prevalence of TB and/or HIV and had implemented TB household contact tracing programs by ward-based outreach teams in line with South Africa’s National Department of Health guidelines. Districts were chosen to represent urban (Ekurhuleni, Gauteng), peri-urban (Bojanala, North West), and rural (uMkhanyakude, KwaZulu-Natal) contexts. Participant groups were chosen to provide an understanding of TB contact tracing from different perspectives and levels of care.

A community stakeholder focus group was held in uMkhanyakude, KwaZulu-Natal only to reflect the tribal community structures in this rural area. Community stakeholders, individuals perceived to represent the community in which TB contact tracing was being implemented, were purposively sampled and recruited for a semi-structured focus group discussion. This focus group was conducted at a neutral and convenient location for all participants.

Individuals over 18 years of age with at least one month of work experience providing facility outreach activities at a household level as either a community care giver, a community health worker, a ward-based outreach team member, or a ward-based primary healthcare outreach team member were classified as ward-based outreach team members for purposes of this study. Ward-based outreach team members were selected by convenience sampling, identified by TB programme stakeholders, such as clinic managers and clinicians, and invited for a semi-structured focus group discussion. Focus groups were conducted at a neutral and convenient location for all participants and held only once, except for in uMkhanyakude, KwaZulu-Natal where two separate focus groups with different ward-based outreach team members were held because of the larger number of interviewees.

Adults, 18 years and older, with TB were purposively selected to include individuals of different ages, genders, and types of TB. People with TB were recruited by a TB nurse during a routine clinic visit. If the TB patient agreed to participate in the study the TB nurse referred them to a study assistant for a comprehensive explanation of the study and to schedule a time for an in-depth semi-structured interview. Since adults with TB were accessed from primary health care clinic records, only individuals attending public health clinics and accessing first-line TB treatment for drug-susceptible TB were included.

The head of household for each person with TB was purposively sampled as the household contact. If the person with TB was the head of the household, the next oldest household member was invited to join the study and scheduled for an in-depth semi-structured interview. Interviews with people with TB and household contacts were conducted in private at their homes or at a location suggested by the participant.

Participant recruitment continued until the desired sample size was reached. If an individual declined participation or repeatedly failed to keep scheduled interview times another individual was purposively recruited. All focus groups and interviews were held face-to-face consisting of a researcher, note taker, and participant/s. Focus group participants were reimbursed ZAR100 (approximately USD10) for time and travel costs. Refreshments were distributed during the community stakeholder focus group discussion.

All interviews and focus groups lasted approximately an hour. Interviews and focus group discussions were digitally recorded, transcribed, and translated into English, then quality checked by a second translator. After interviews and focus groups, interviewers reconvened to discuss and learn from each other’s experiences. Transcripts were reviewed and participant-identifying information was removed. Participants did not provide feedback or review transcripts.

Interviewers were fluent in English and either isiZulu, isiXhosa or Setswana as interviews were conducted in the language preferred by the participant. Three co-investigators, two co-ordinators and fourteen research assistants, a mixture of education levels, genders, and ages, conducted interviews. Investigators held a PhD and/or a medical degree, coordinators held a master’s and/or bachelor’s degree, and research assistants had at least two years of qualitative data collection experience. Interviews and focus group discussions were semi-structured, therefore interview guides were developed, drawing from the Consolidated Framework for Implementation Research and relevant literature, to ensure consistency in data collection across districts [20].

### Ethics

All participants provided written consent prior to any engagement in research. The University of the Witwatersrand Human Research Ethics (ref. HREC 160,305), the Biomedical Research Ethics Committee of the University of KwaZulu-Natal (ref. BE246/16), and the London School of Hygiene & Tropical Medicine (ref. 11,020) provided ethics permissions. The Department of Health in KwaZulu-Natal (HRKM 353/16), Gauteng (GP-2016RP1_403), and North West Province (NW_2016RP59_358) all provided district approvals.

### Analysis

Thematic analysis was performed to extract broad themes from a subset of all transcripts. Themes were refined through the iterative process of reading and manually coding. Broad themes and potential subthemes appearing across transcripts were consolidated into a codebook. The codebook was independently applied to a selection of transcripts by a senior social scientist (CMC) to assess intercoder reliability and saturation of themes. Themes lacking reliability were discussed until consensus was met and the codebook was refined. Through deductive coding we focussed on four key categories: drivers and facilitators of stigma, stigma marking, manifestation, and outcomes of stigma, as per the health stigma and discrimination framework [6]. Further development of themes and subthemes were made through the iterative and inductive process of coding until no further subthemes could be identified. QSR NVIVO was used to apply the final codebook across all 48 transcripts, including those previously manually coded, leading to the finalization of themes and aggregation of supporting quotations.

## Results

Data collection for this study was conducted between May 2016 and February 2017. Table 1 displays the number of interviews, focus group discussions, and participants by district and participant category. Of the 94 participants included in the analysis, 31 (13 female) were people with TB, 12 (5 female) were household contacts, 40 (39 female) were ward-based outreach team members, and 11 were community stakeholders. Table 2 displays themes and their definitions.

**Table 1:** Enrolled participants and interviews/focus group discussions by district

	Ekurhuleni, Gauteng	Bojanala, North West	uMkahanyakude, KwaZulu-Natal	Total
TB Index Patient Interviews	9	10	12	31
Household Contact Interviews	3	4	5	12
Ward-based Outreach Team Focus Group Discussions	1	1	2	4
Ward-based Outreach Team Focus Group Participants	10	12	18	40
Community Stakeholders Focus Group Discussions	0	0	1	1
Community Stakeholders Focus Group Participants	0	0	11	11
Total Participants	22	26	46	94

**Table 2:** Domains, themes, and definitions

Domains^1^	Themes	Definitions
Drivers & Facilitators	Fear of association with HIV	Fear of getting or being identified as having HIV
		Widespread community understanding of HIV and TB coinfection
	Fear of TB transmission	Fear of transmitting TB to others and/or getting TB
Stigma Marking	Verbal markers of TB leading to stigma	Gossip about TB status in the community and at clinic
		Desire for confidentiality when testing
	Visual markers of TB leading to stigma	Display of TB symptoms
		Use of TB services at facilities
		Possession and use of medication
		Household visits by ward based outreach teams
Manifestations	Anticipated Stigma	Low self esteem and self worth
		Discreditation of character & social undesirability
	Internalized Stigma	Application of anticipated stigma to self
Outcomes	Avoidance of stigma marking	Avoidance of clinic to avert visual and verbal stigma marking
		Rejection of ward based outreach teams to avert visual and verbal stigma marking
	Desire for education	Education on TB transmission and prevention
		Education on stigma and how to communicate with people with TB

^1^ Domains align with the health stigma and discrimination framework to enable a cross cutting socio-ecological framing of results.

### Theme 1: fear of association with HIV

Interviewees from all participant groups understood TB to be a marker for HIV infection and illuminated that both HIV and TB inspired fear and stigma within the community. People with TB and their household contacts feared getting and/or being identified as having HIV.

“Ahh that [referring to encouraging family members to test for HIV] won’t come from me because when someone tests and find that he is positive and he will blame me and say I said he must test and he will hate me with all his life.” Interview, Person with TB, Ekurhuleni.

“They can screen you for TB but there are those diseases that people fear the most like HIV.” Interview, Person with TB, uMkhanyakude.

The comorbidity of HIV and TB disease was widely understood by study participants, enabling TB to be a potential marker for HIV and their associated stigmas.

“They spoke on radio they say that it’s something that goes hand in hand you see, they also say that before being initiated on [HIV] treatment after you discovered that you have HIV you should also check for TB, so it’s very important.” Interview, Household contact, uMkhanyakude.

“I think HIV and TB they are they almost they almost [sic] working the same stream, so if you have TB it’s easy so if have HIV it’s easy to have TB [sic]” Interview, Person with TB, Bojanala.

### Theme 2: fear of TB transmission

People with TB feared transmitting the disease to others and the detrimental effects of disease.

“That when I eat I must eat alone not with any person, even young infant… There’s an young kid young young young [sic] and she says I am not to hold because she will be infected” Interview, Person with TB, Ekurhuleni.

“I was concerned…I isolated myself from them I sat alone at the corners [pause] afraid of infecting them” Interview, Person with TB, Bojanala.

Community stakeholders, ward-based outreach team members, and household contacts of people with TB reported feeling at risk of infection.

“First thing I do [is] I knock at the door and then you answer. It’s early in the morning. The windows are still closed. I’m going [to] ask you to open the windows because for us, it’s just risky to just enter.” Focus group discussion, Ward-based outreach team member, Ekurhuleni.

“We have someone who has TB in the house, we sometimes think that we might be infected because we have not got a treatment or being told to visit the clinic to get tested for TB or even to prevent being infected.” Interview, Household contact, Bojanala.

### Theme 3: verbal markers of TB leading to stigma

People with TB, household contacts, and ward-based outreach team members noted that individual’s disease status could be identified, without disclosure, through gossip. Some community members felt that ward-based outreach team members would gossip with community members disclosing disease status without consent.

“I had a fight with someone who is in community care giver [ward-based outreach team member] and she or he come to visit me because he or she’s her work neh [sic] and when she come here she found that I’m I have TB or low blood any disease that he come here with it then she or he go to the to someone else tell that person about my status or my disease yah“ Interview, Person with TB, Bojanala.

“The problem is that community care givers [ward-based outreach team members] they do not have secrets of the people [confidentiality], the clinic is alright because community caregivers [ward-based outreach team members] do not keep secrets of the people, like to say, they come to check here at home and then they go to that school - at the end you hear bad talks about you there is no way you cannot be hurt, yes.” Interview, Person with TB, uMkhanyakude.

Other community members felt that gossip occurred when accessing facility-based care.

“it will be better if checked at homes because here in clinic it is worrying…it worries us because [inaudible segment] here you are being checked and there are your relatives…others you find that if you have a problem, it [one’s diagnosis] will spread all over the place.” Interview, Person with TB, uMkhanyakude.

People with TB and their household contacts expressed distrust in patient confidentiality due to gossip when receiving care by individuals living within their community.

“Something bad is that when a person is a [ward-based outreach team member] and these we live with those people in the community…Then, you will do all these things and he/she will take your whole information you see…He/she will gossip about that thing…it will really affect you as a citizen.“ Interview, Household contact, uMkhanyakude.

### Theme 4: visual markers of TB leading to stigma

People with TB, household contacts, and ward-based outreach team members felt that TB disease status could be visually identified when attending clinics. Most facilities have a separate area where people with TB receive care. The division of patients can indirectly disclose disease status and encourage gossip.

“I can walk with my neighbour, when we arrive at the gate, we take the treatment up there and everyone knows that treatment is taken up there then we depart. She will go down there at the clinic and I will go up there. She can gossip about me taking TB and HIV treatment because they know that TB and HIV are interrelated.” Interview, Person with TB, uMkhanyakude.

“People talk…that is how news travel fast because they see a person at the clinic in a queue for treatment then they start to gossip about the person, then the whole community knows about the person’s status” Interview, Household contact, Bojanala.

Symptoms of illness facilitated the identification and marking of community members. People with TB and their household contacts identified common symptoms such as coughing, weakness, and weight loss.

“Apart from coughing you see, such a person will become weak, you will hear him/ her saying he/she does not have strength, he or she likes to cover himself/herself [cover with blanket] even when it is hot during the day like this he/she would be wearing a big [cover] you see, he or she is losing weight therefore he or she is hiding the body.” Interview, Person with TB, uMkhanyakude.

Household visits by ward-based outreach teams were perceived to mark homes as belonging to the chronically ill leading community members to assume a sick person lives there.

“Oh, I was scared [when a ward-based outreach team came to my house], but there was nothing I can do, I couldn’t run away from them. Yes, I was so scared hey… plus most of the times when people from the clinic come, people will start judging me.” Interview, Person with TB, Bojanala.

“There are people in the community who don’t like when we go in [the community]. [They are] thinking that neighbours will say this and say that.” Focus group discussion, Ward-based outreach team member, Ekurhuleni.

People with TB and their household contacts felt that their disease state could be identified through the ownership and use of medication as well as through the length of treatment.

“No it is that…you see, I see everyone at home taking pills… one is taking them continuously [life time treatment] and the other is taking… something that ends [short period treatment] you see, there is… there is discrimination there you see, many things…things like that.” Interview, Person with TB, uMkhanyakude.

“Yes, neighbours are judgemental saying” mm…mm what is she diagnosed for? Maybe she’s on that treatment what…what? That 8 o’clock cornmeal… maybe she’s taking that 8 o’clock corn meal [to prevent treatment nausea]? As you see people from the clinic maybe she has defaulted” [sic]” Interview, Person with TB, Bojanala.

### Theme 5: anticipated stigma

Participants reported anticipated stigma.

“Meaning at that time some people who would be looking at me at a distance, from other communities, the community will say that area is full of that thing [TB]. So the integrity of a person is lost and the dignity of the community is also lost.” Interview, Person with TB, uMkhanyakude.

“Others are afraid that I will not be accepted by girls that I am sick, others are afraid that I will not be approached by men because I am sick” Interview, Person with TB, Bojanala.

### Theme 6: internalized stigma

Internalized stigma manifested when people with TB accepted the negativity that they encountered and turned these feelings inwardly.

“One which is now a problem is TB because it is not easy to control you see. If I come to them, they stand up and go and I end up sitting alone, what do I do after that, I then see that which means that, as people do not want me, they run away from me, why am I living, I then hang myself up” Interview, Person with TB, uMkhanyakude.

“A person with TB because if you discriminate him [sic] he will never heal because his heart will always think that now they are discriminating me because there is no where you can put him you cannot throw them away.” Interview, Household Contact, uMkhanyakude.

### Theme 7: avoidance of stigma marking

Community perceptions of TB stigma impacted if, where, how, and what health services were accessed. TB patients reported avoidance of seeking care in settings where they could be potentially marked with stigma.

“People do not want to come to the hospital my sister, anyone especially we males do not want to come to the hospital. We are afraid of what others will say and the queue. We are afraid the queue… women… busy queueing with women, causing traffic like that you see. What other people in the area will say after I was seen at the clinic, that is why I say if we can get special places that are available, that are hidden like… that’s what I can say.” Interview, Person with TB, uMkhanyakude.

Many individuals reported a preference for seeking care at clinics due to the potential for stigma marking of those living in homes visited by ward-based outreach teams.

“Yes [Ward-based outreach team members] gossip but if you just go there yourself to the health department it becomes a quiet thing you can find that as I am with you here. You can find that you have TB and when you do that thing it will end there” Interview, Household contact, uMkhanyakude.

“I don’t trust the community givers [Ward-based outreach team members] I prefer the clinic” Interview, Person with TB, Bojanala.

Conversely, many individuals reported a preference for home-based services due to potential for stigma marking when accessing facility-based care.

“Some of us cannot stand the crowd at the clinic, looking at the fact that some people will be looking at you and want to know why you are coming to the clinic, what test you want to do or maybe you’re already infected, then they start to look at you in a different way” Interview, Household contact, Bojanala.

“It is very simple because I think it can be a household secret. [At the clinic] it won’t be a secret if you are afraid that other people will see that oh, he/she went into that room where they test people who are like this. So if it is at home it will be easy if everything is done at home.” Interview, Person with TB, uMkhanyakude.

### Theme 8: desire for education

People with TB and their household contacts believed that community level education would promote understanding of TB infection, how to communicate with people with TB, and how to prevent the perpetuation of stigma against people with TB.

“People are afraid you see, for instance they will always say to someone [with TB] he/she must lock him/herself in a room you see… I think that [education] could help us to avoid discriminating that person with TB.“ Interview, Household Contact, uMkhanyakude.

“You can tell people about sicknesses […], you have been told and trained without seeing a person walking along the way that […], seems like they have TB hhayi [sic] [no] it is not easy that but if you are trained and [know] how to approach a person so that they do not fight and get angry at you, talk to them properly, [you] need to be trained how to talk, need to be trained [how] to approach a person, trained [how] to show a person until they get on track that hhayi [no] truly this thing will help me tomorrow” Interview, Person with TB, Bojanala.

## Discussion

Our results support and build upon literature on TB stigma in Southern Africa by describing themes surrounding TB stigma and aligning them with the health stigma and discrimination framework [6]. Understanding how TB stigma functions in the community can inform targeted interventions and approaches to dismantle or disturb processes contributing to TB stigmatization and to improve TB case finding, treatment, and retention in care.

We found that fear of TB infection/transmission and TB/HIV stigma are integral to driving and facilitating TB stigma. Fear of infection, as well as transmission, has been repeatedly documented in social theory and in TB stigma studies across varying contexts, including South Africa [12, 21–28]. The high prevalence and widespread understanding of HIV and TB in South Africa contribute to the construction of a double TB HIV stigma [14, 16, 17]. Research on this double TB HIV stigma in South Africa shows that TB patients attempt to create distance from HIV stigma through covering or deflecting attention away from their HIV status and onto their TB status [16, 17]. Similarly, we found that patients differentiated between their diagnoses and avoided engaging in conversation about HIV, despite widespread understanding of TB and HIV infection. Further research on the double stigma of TB and HIV followed by sensitivity training of clinical staff could help to combat TB stigma. Improving community knowledge about TB and its transmission could reduce the fear that drives and facilitates TB stigma. Community members voiced that they were concerned about TB transmission, yet many people did not understand that once a patient is on treatment, they are no longer able to transmit TB. An education campaign communicating the message, if on treatment there is no transmission, similar to the HIV campaign Undetectable = Untransmissible (U = U), could decrease stigma while strengthening active case finding, treatment, and retention in care.

We found that stigma marking took place verbally, through gossip, as well as visually, through symptoms and/or along the pathway to care. Gossip about TB status has been repeatedly reported as a method to identify a patient’s disease status without their disclosure [26, 29–31]. A study in South Africa similarly documented that the visual identification of symptoms such as, coughing, weight loss, sweating, and diminished appetite were identifiers for TB and/or HIV [18]. An evaluation of a contact tracing program in South Africa similarly noted that community members found ward-based outreach teams to be stigmatizing due to their association with disease, highlighting that some community members chased ward-based outreach teams off their property [32]. A study in Zambia and South Africa on HIV found that patients’ status was identified through queues when accessing care at clinics [33]. Similarly, we found that provision of care at the clinic and/or at home marked those receiving care with TB stigma. A South African study on TB patients who were also HIV-positive, found that patients hid their medication to avoid associated stigma, confirming our findings that medication has the capacity to mark patients with stigma [15]. Training through the health system on stigma, discrimination, privacy, and patient confidentiality, especially within community and facility-based models of prevention and care, could have benefits in reducing TB stigma. Clinics that provide disease-specific services need to do so in a way that does not disclose disease status. Education campaigns teaching individuals that early testing and treatment reduces disease severity could lead to more individuals testing for TB earlier to avoid symptomatic identification and reduce community fear while increasing case identification, treatment, and retention in care. Further research is required on how best to combat gossip within communities. A potential approach could be illuminating that gossip not only hurts the individual but also the reputation of the community.

The manifestation of stigma as anticipated, enacted, and/or internalized are repeatedly documented within academic literature across varying contexts amongst TB patients and healthcare workers [8, 34]. We found that anticipated and internalized stigma emerged as themes. Unfortunately, the literature on stigma within Southern Africa is sparse with inconsistent operationalization of stigma terminology [34]. Studies in South Africa and Zambia confirm that internalized stigma is marked by low self-worth and that anticipated stigma impacts disclosure of one’s status due to the fear of negative reactions [18, 35]. Two South African studies investigated TB and HIV stigma and similarly reported the presence of anticipated stigma marked by negative associations of TB and that individuals with internalized stigma had low self-esteem [17, 36]. The presence and manifestation of stigma as anticipated and internalized aligns with our findings. People with TB and their household contacts mentioned that education on best practices for recovery through treatment from TB survivors would be helpful as TB survivors are living proof that TB and the associated stigma can be overcome, this also has the potential to bolster treatment adherence and retention in care.

We found that there was a need for community and individual education on how to prevent TB transmission, promote caring for people with TB, and combat stigmatization of TB within the community. This was also found within other investigations in South Africa and Sub-Saharan Africa [14, 37, 38]. We found that the avoidance of TB stigma, or stigma mediation, impacted the way in which individuals accessed healthcare. Stigma mediation was highly individualized and dependent upon ones understanding of and experience with stigma. This manifested through contrasting views as some community members felt stigma marking took place at facilities and others with ward-based outreach teams. Similarly, other South African studies found that individuals’ perceptions of stigma marking impacted how they accessed health services, such as preferences for home-based care over facility-based care or, not presenting at all [14, 15, 39]. This individualized navigation of services highlights the need for a person-centred approach to combat stigma, rather than an all-encompassing approach. Sensitivity training for ward-based outreach team members and client facing facility staff on stigma could reduce the stigmatization felt by people with TB and their household contacts during household visits or when visiting the clinic. Community members requested TB education and believe in its capacity to reduce TB stigma. With this support, community education in this context could be an efficacious anti-stigma intervention.

There is a dearth of research on TB stigma reduction strategies. A 2017 systematic review on evidence-based interventions to reduce tuberculosis stigma found only seven studies, two of which were conducted in sub-Saharan Africa [40]. Studies included in the systematic review focused on TB education campaigns to reduce stigma [40]. Intervention outcomes were contradictory as one South African study found that TB stigma increased with accurate knowledge of TB transmission, conversely, a study in Ethiopia showed a decrease in stigma as knowledge of TB increased [41, 42]. This conveys that anti-stigma interventions, especially education campaigns need to be executed with precision and care, with an understanding of how TB stigma functions at a community level to efficiently deconstruct processes and/or appropriately intervene. A 2022 scoping study on interventions designed to reduce tuberculosis-related stigma could only draw from nine articles, reconfirming the dearth of literature [34]. Further they highlighted inconsistent stigma definitions, a need for standardized measurement, and more measurement of stigma intervention outcomes [34].

### Conclusion

We found that TB stigma exists at a community level in South Africa. It is driven and facilitated by fear of infection and illness coupled with knowledge of TB/HIV duality and manifests as internalized and anticipated stigma. Disease status, and accompanying stigma marking, can be surmised visually, when accessing care at public health clinics and/or through ward-based outreach teams, through treatment, and symptoms, as well as verbally, through gossip. People with TB try to avoid stigma marking when accessing clinical care which limits access to and provision of TB services. Further research is required to understand how to respond to TB stigma carefully and responsibly as interventions could potentially contribute to stigmatization.

## Electronic supplementary material

Below is the link to the electronic supplementary material.

INTERVIEW GUIDE: HOUSEHOLD CONTACTS

INTERVIEW GUIDE: PEOPLE WITH TB

## Data Availability

The datasets generated and/or analysed during the current study are available from the corresponding author on reasonable request.

## List of Abbreviations

TB: Tuberculosis
