# Supplementary material for: A qualitative exploration into the presence of TB stigmatization across three districts in South Africa
Source: BMC Public Health. 2023 Mar 15;23:504. doi: 10.1186/s12889-023-15407-2 (PMC10017062; doi:10.1186/s12889-023-15407-2)
Supplement: Supplementary file 1 — INTERVIEW GUIDE: HOUSEHOLD CONTACTS [file 12889_2023_15407_MOESM1_ESM.doc]

### INTERVIEW GUIDE: HOUSEHOLD CONTACTS

## OPTIMIZING THE EFFICIENCY OF HOUSEHOLD CONTACT TRACING FOR TB CONTROL IN SOUTH AFRICA

#### Introduction and Ground Rules

1. Obtain written informed consent first, before any data are collected.
2. Interviewer to introduce self. Thank you for taking the time to meet with us today. Our names are [*insert names*] _____________ and we would like to talk to you about TB contact tracing. We are doing this project to understand how we might improve TB contact tracing in the community, and particularly to see whether there are better ways to deliver contact tracing. We are interested in your views regardless of whether you have or have not got experience of TB contact tracing. We want you to be open when answering. There are no right or wrong answers in this discussion. Please feel free to tell us what you think.
3. Interviewer to explain the ground rules and terms of confidentiality for the interview:
   - The participant does not have to answer any question they do not want to.
   - The information you share will be handled in confidence. (in secret)
   - When we report back on the information collected in this discussion, your comments will not be able to be linked to you specifically.
   - We ask that you also agree not to share anything discussed in this room with others.
4. The discussion should take around one hour.
5. Interviewer to inform the interviewee that the in-depth interview will be tape recorded to make sure that all themes are captured. Turn the tape on and ask for verbal permission again to tape record, while the tape is running to verbally capture consent (this is a double check against the written consent). We will be recording the session because we don’t want to miss any of your comments. Although one of us may take some notes while we talk, we can’t write fast enough to get everything down on paper. As we are recording, please try to speak loudly so that we don’t miss your comments.

#### Themes to be explored

1. Knowledge and experience of TB (including TB contacting tracing and acceptability)
2. Knowledge and experience of HIV (including HIV counselling and testing and preference for HIV counselling)
3. Preference for any additional health services during a contact tracing visit

#### Time started (HHMM):

#### Questions

- 1. **Knowledge and experience of TB**
     - *Have you heard about a disease called TB (tuberculosis)? Can you explain to us the type of information on TB that you heard about?*
     - *Do you know how tuberculosis can be spread? If yes, tell me more.*
     - *Can you talk about TB in your house? If yes or no, what are some of the reasons?*
     - *Have any health workers visited you since someone in your house was diagnosed with TB? What did you like/dislike when your house was visited?*
     - *How could we make it better for you or other people in your community when health workers visit you?*
     - *What do you think your neighbours would say if they saw a health service vehicle at your house? Does this make you feel good or bad? Would it be better or worse if the vehicle didn’t have signs on it?*
  2. **HIV testing**
     - *Tell me what you know about HIV and about how it affects your community?*
     - *Describe your experience of being tested for HIV? (Probe on where and when HIV testing took place)*
     - *How do you feel about health workers offering you HIV testing in your house? Please tell why you would choose this as an option or not support this method of testing*
     - *What do you think about HIV testing being done at the same time as being checked for TB?*
     - *Did you test for HIV recently? Can you please describe to us what factors influenced your decision to test for HIV?*
     - *How did you feel about the process?*
     - *Were there any difficulties with HIV testing in your house?*
     - *Is there anything you think could be done differently when health workers visit your house?*
  3. **Preferences for contact tracing**
     - *Please describe your experience of TB contact tracing*
     - *Would you have preferred to go visit a clinic or have health workers visit you at home; what are some reasons for saying this?*
     - *What do you understand by the term ‘community care giver’?*
     - *Can you tell me about any experience you have of being visited by a community care giver?*
     - *What would you feel about a community care giver being the person that checks the family for TB and HIV?*
     - *When is the best time for us to visit you at your home to provide health services? During the day or in the afternoon after work?*
     - *When health staff visit you at home, are there any other things you would like them to help you with? Blood pressure or diabetes check?*
  4. Would you allow health workers to visit your household again in the future for household contact tracing of TB? Can you explain your answer?

***Any other comments***

Are there any final thoughts you have about TB household contact tracing?

***End of session***

Now we have come to the end of our discussion. Thank you for your participation. If you have any questions about your study participation, please contact us. Thank you.

#### Time ended (HHMM):
