# Supplementary material for: A qualitative exploration into the presence of TB stigmatization across three districts in South Africa
Source: BMC Public Health. 2023 Mar 15;23:504. doi: 10.1186/s12889-023-15407-2 (PMC10017062; doi:10.1186/s12889-023-15407-2)
Supplement: Supplementary file 2 — INTERVIEW GUIDE: PEOPLE WITH TB [file 12889_2023_15407_MOESM2_ESM.doc]

### INTERVIEW GUIDE: PEOPLE WITH TB

## OPTIMIZING THE EFFICIENCY OF HOUSEHOLD CONTACT TRACING FOR TB CONTROL IN SOUTH AFRICA

#### Introduction and Ground Rules

1. Obtain written informed consent first, before any data is collected.
2. Interviewer to introduce themselves: Thank you for taking the time to meet with us today. Our names are [*insert names*] _________), and we would like to talk to you about TB contact tracing. We are doing this project to understand how we might improve TB contact tracing in the community, and particularly to see whether there are better ways to deliver contact tracing. We are interested in your views regardless of your experience with TB contact tracing. We want you to be open when answering. There are no right or wrong answers in this discussion. Please feel free to tell us what you think.
3. Interviewer to explain the ground rules and terms of confidentiality for the interview:

- The Participant does not have to answer any question they do not want to.
- The information you share will be handled in confidence (in secret).
- When we report back on the information collected in this discussion, your comments will not be able to be linked to you specifically.
- We ask that you also agree not to share anything discussed in this room with others.

1. The discussion should take around one hour.
2. Interviewer to inform the interviewee that the in-depth interview will be tape recorded to make sure that all themes are captured. Turn the tape on and ask for verbal permission again to tape record, while the tape is running to verbally capture consent (this is a double check against the written consent). We will be recording the session because we don’t want to miss any of your comments. Although one of us may take some notes while we talk, we can’t write fast enough to get everything down on paper. As we are recording, please try to speak loudly so that we don’t miss your comments.

#### Themes to be explored

1. Knowledge and experience of TB (including TB contacting tracing and acceptability)
2. Knowledge and experience of HIV (including HIV counselling and testing and preference for HIV counselling)
3. Preference for any additional health services during a contact tracing visit

#### Time started (HHMM):

#### Questions

1. Can you tell me what you understand about how TB spreads from person to person?
2. When you were diagnosed with TB, were you worried about the health of your household members?
   - *How important do you feel it is for your family to be checked for TB?*
   - *Could you explain your answer?*
3. Could you describe whether anything happened for your household members after you were diagnosed with TB?
   - *Did they receive a visit at home to check them for TB?*
   - *Or were they advised to visit a clinic or hospital to be checked for TB?*
4. If there are children at home, can you describe whether anything happened specifically for the children?
   - *Did any of the children get tested for TB at home?*
   - *Did any of the children go to the clinic or hospital to be tested for TB?*
5. How important do you think it is for household members to be checked for HIV when someone is diagnosed with TB?
   - *Why is it important?*
6. Can you explain whether your family has had any checks for HIV since you were diagnosed with TB?
   - *If not, would you have liked your family to be checked for HIV?*
7. Where do you think these checks (for TB and HIV) should take place?
   - *Would it be better to happen at home, or at the clinic or hospital?*
8. Do you have any experience of having been visited at home by community care givers?
   - *If yes, can you tell me more?*
   - *What did the community care giver do during the visit?*
9. What would you feel about community care givers coming to the home to check people for TB and HIV?
   - *Would it be acceptable for a community care giver to visit the home for this?*
   - *What concerns would you have about a community care giver doing this?*
   - *Would you prefer for people focused on TB to visit your family at home?*
10. Other than checking the family for TB and HIV, is there anything else that you think it would be important for them to be checked for?
11. Are there any other health care services that should be provided for your family at this time?

***Any other comments***

Are there any final thoughts you have about TB household contact tracing?

***End of session***

Now we have come to the end of our discussion. Thank you for your participation. If you have any questions about your study participation, please contact us. Thank you.

#### Time ended (HHMM):
